# Global trends and hotspots in diabetes and hearing loss: a 20-year systematic bibliometric study

**DOI:** 10.3389/fmed.2026.1867924

**Published:** 2026-07-28

**Authors:** Zhaoquan Yi, Xinying Ni, Mingxiao Guo, Hua Shao, Xuelei Chen, Fang Zhang

**Affiliations:** 1 Shandong Academy of Occupational Health and Occupational Medicine, Shandong First Medical University and Shandong Academy of Medical Sciences, Jinan, China; 2 Dezhou Center for Disease Control and Prevention, Dezhou, China; 3 Monitoring and Evaluation Center, Shandong Academy of Occupational Health and Occupational Medicine, Shandong First Medical University and Shandong Academy of Medical Sciences, Jinan, China

**Keywords:** bibliometric analysis, cognitive impairment, diabetes mellitus, hearing loss, knowledge mapping, older adults, precision medicine, sensorineural hearing loss

## Abstract

**Background:**

Diabetes and hearing loss are increasingly recognized as critical global public health concerns. Cumulative evidence indicates a close association between diabetes and auditory impairment; however, the knowledge structure, research hotspots, and developmental trends in this field remain insufficiently and systematically characterized. This systematic bibliometric review aims to comprehensively map the global research landscape, collaboration patterns, knowledge structure, thematic evolution, and emerging research frontiers regarding diabetes and hearing loss over the past two decades.

**Methods:**

A systematic bibliometric review was conducted on publications related to diabetes and hearing loss. Relevant studies published between 2006 and 2025 were systematically retrieved from the Web of Science Core Collection and Scopus databases based on predefined search strategies and eligibility criteria. After database merging, deduplication, and eligibility assessment, a total of 1,622 publications were included in the final analysis. VOSviewer, CiteSpace, and Bibliometrix were employed to analyze annual publication output, collaboration networks, core journals, keyword co-occurrence, thematic evolution, co-citation patterns, and citation bursts.

**Results:**

Research on diabetes and hearing loss presents a sustained upward growth trend and has gradually evolved into a multidisciplinary field covering otology, endocrinology, neurology, geriatrics, genetics, and molecular biology. The United States occupies a core position in the global research collaboration network, while institutions across North America and Europe contribute predominantly to knowledge production in this domain. Keyword and citation burst analyses identify hearing loss, diabetes mellitus, sensorineural hearing loss, and Wolfram syndrome as the core research topics of the field. Research priorities have progressively shifted from investigations on syndromic and mitochondrial disorders to explorations of age-related hearing loss, modifiable risk factors, cognitive impairment, older adult populations, precision medicine, and inflammation-associated mechanistic mechanisms.

**Conclusion:**

Over the past two decades, research on diabetes and hearing loss has achieved substantial expansion and continuous interdisciplinary integration. The research field has transitioned from early focuses on syndromic and mitochondrial disorders to a broader and more clinically practical framework that covers epidemiological associations, risk stratification, aging, cognitive impairment, and precision medicine. These findings clarify the developmental trajectory and emerging research priorities of this field and can guide future mechanistic research, early screening formulation, and targeted intervention development.

## 1 Introduction

Diabetes mellitus (DM) is one of the most common chronic metabolic diseases worldwide (1). Its prevalence continues to rise, rendering it a major public health concern (2). Chronic hyperglycemia can damage multiple organ systems and trigger a range of chronic complications. In addition to the retina, kidneys, peripheral nerves, and cardiovascular system, a growing body of evidence indicates that the auditory system may also serve as a primary target organ for diabetes-induced damage (3, 4).

Hearing loss is a prevalent sensory impairment that substantially affects individuals’ communication abilities, social participation, quality of life, and cognitive function (5). In recent years, the potential association between diabetes and hearing loss has garnered extensive research attention. Multiple epidemiological studies have suggested that individuals with diabetes exhibit an elevated risk of hearing loss, especially among middle-aged and older adults. Existing research demonstrates that microvascular complications, oxidative stress, chronic inflammation, mitochondrial dysfunction, and neurodegenerative changes collectively contribute to the pathogenesis of diabetes-related hearing loss (3, 6). However, current evidence remains heterogeneous, and consistent conclusions regarding study populations, evaluation criteria, underlying mechanisms, and clinical significance have not been reached across studies (7).

With ongoing advances in scientific research, the body of literature investigating the relationship between diabetes and hearing loss has expanded rapidly. The research scope has gradually broadened from early focuses on syndromic and mitochondrial disorders to cover epidemiological characteristics, risk factors, age-related hearing impairment, cognitive dysfunction, and precision medicine (8). Meanwhile, this research field exhibits distinct interdisciplinary features, encompassing otolaryngology, endocrinology, neurology, geriatrics, molecular biology, and other related disciplines (9). Although previous studies have explored the correlation between diabetes and hearing loss from diverse perspectives, systematic summaries regarding the overall research status, core research topics, and evolving trends in this field remain scarce. As a quantitative analytical method for academic literature, bibliometrics adopts statistical and visual analytical approaches to identify research trends, knowledge structures, hot topics, and cutting-edge research directions in a specific field (10). Compared with traditional narrative reviews, bibliometric analysis not only enables systematic evaluation of research outputs but also reveals the evolutionary patterns of research topics and academic collaboration networks through keyword co-occurrence analysis, co-citation analysis, and collaborative network construction (11, 12). The application of bibliometric methods to diabetes and hearing loss research facilitates a comprehensive understanding of the overall research landscape and identifies promising directions for further investigation. Therefore, this study conducted a systematic bibliometric review of publications on diabetes and hearing loss published from 2006 to 2025. By implementing predefined, reproducible literature retrieval strategies, explicit eligibility criteria, standardized study selection procedures, and multidimensional bibliometric and visual analyses, this review aims to characterize the global publication profile, academic collaboration patterns, intellectual structure, thematic evolution, and emerging research frontiers in this interdisciplinary field.

## 2 Materials and methods

### 2.1 Study design and reporting framework

This study adopted a systematic bibliometric review and visualization analysis design. Unlike conventional systematic reviews that synthesize clinical effect estimates, the present review systematically identified, screened, and mapped relevant literature to characterize publication patterns, collaboration structures, intellectual foundations, thematic evolution, and emerging research frontiers. Accordingly, this review integrated a structured literature search and study selection process with quantitative bibliometric and visualization analyses. The literature search and study selection procedures were reported in accordance with the PRISMA 2020 statement, which was used as a guiding reporting framework to ensure transparency and completeness of the review process and the search strategy was further documented following the PRISMA-S extension. Since this review aimed to map publication and citation patterns rather than synthesize clinical effect estimates, conventional meta-analysis and study-level risk-of-bias assessments were not conducted.

### 2.2 Data sources and search strategy

The core research question of this study was defined as follows: What are the publication trends, collaboration patterns, knowledge structures, research hotspots, and emerging frontiers in the field of diabetes and hearing loss research from 2006 to 2025? Web of Science Core Collection (WoSCC) and Scopus databases were selected for analysis due to their broad multidisciplinary coverage, standardized citation metadata, and compatible bibliographic fields for mainstream bibliometric analysis tools. The combined use of the two databases improves literature coverage and mitigates potential biases caused by reliance on a single citation database.

On 25 February 2026, a systematic literature search was performed in both the WoSCC and Scopus databases to retrieve studies focusing on diabetes and hearing loss. The search timeframe was restricted to publications issued between 2006 and 2025, and document types were limited to original articles and review articles. Only English-language publications were included in the analysis to ensure consistency in bibliographic field extraction, keyword standardization, and network construction across the two databases.

The search query applied in WoSCC was as follows:

TS = ((“hearing loss” OR “hearing impairment” OR deafness OR presbycusis) NEAR/20 (diabetes OR “diabetes mellitus” OR “type 2 diabetes” OR “insulin resistance”)).

The search query applied in Scopus was as follows:

TITLE-ABS-KEY((“hearing loss” OR “hearing impairment” OR deafness OR presbycusis) W/20 (diabetes OR “diabetes mellitus” OR “type 2 diabetes” OR “insulin resistance”)). The NEAR/20 and W/20 proximity operators were adopted to capture the contextual co-occurrence of diabetes-related and hearing-related terms, while adapting to the syntactic differences between the two database search systems.

**Figure 1:**
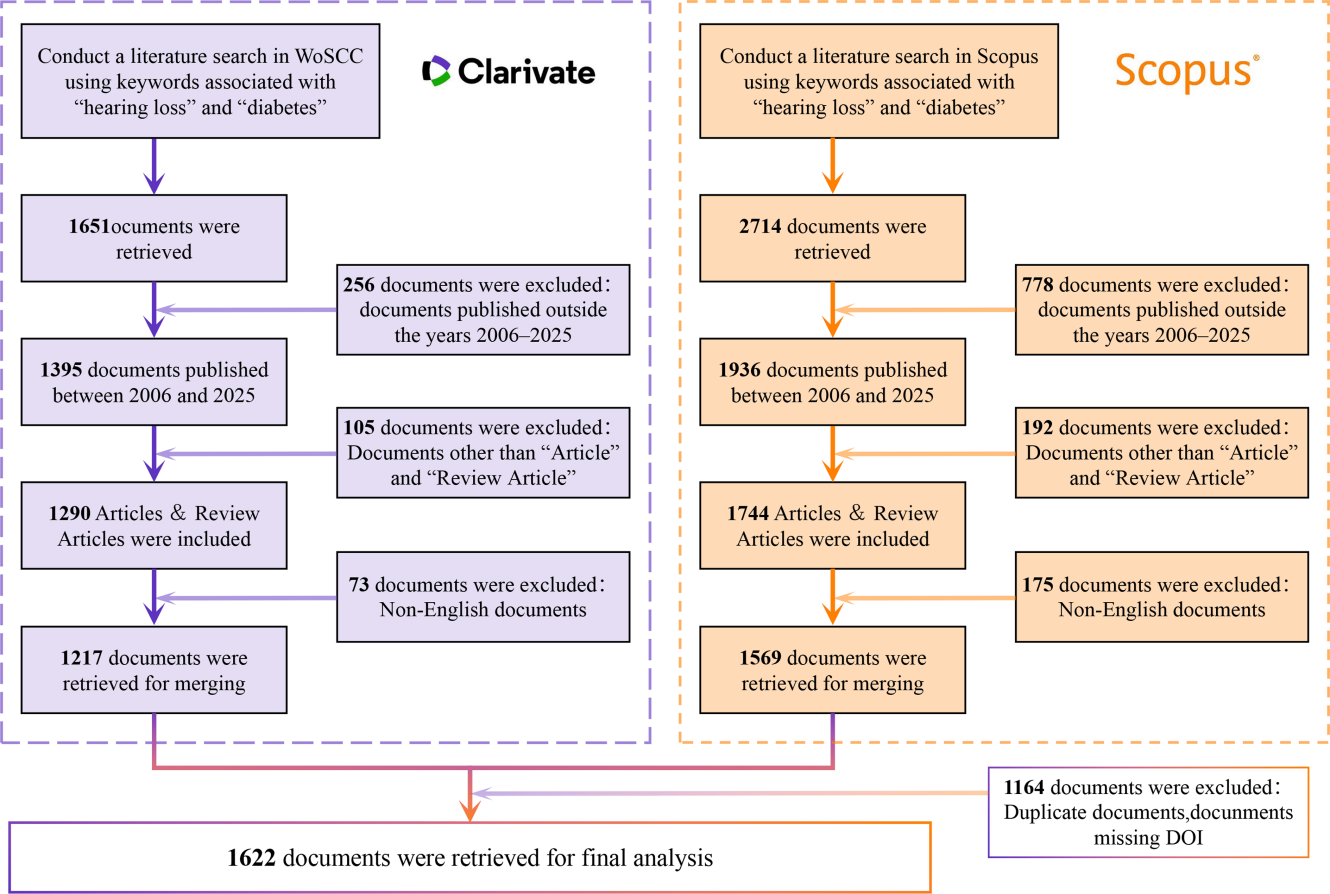
Flowchart of literature retrieval, screening, and selection.

According to the predefined screening criteria, the initial WoSCC search returned 1,651 articles. After excluding 256 articles published outside the 2006–2025 timeframe, 105 non-qualifying documents (non-Article and non-Review Article types), and 73 non-English publications, a total of 1,217 eligible articles were retained for database consolidation. The initial Scopus search yielded 2,714 articles. After excluding 778 articles outside the specified publication timeframe, 192 non-qualifying document types, and 175 non-English articles, 1,569 articles remained for subsequent consolidation. The detailed literature screening and inclusion workflow is presented in Figure 1.

### 2.3 Eligibility criteria, study selection, and data extraction

Publications were included if they: (1) focused on the relationship between diabetes or related metabolic disorders and hearing loss or auditory dysfunction; (2) were categorized as articles or review articles; (3) were published in English between 2006 and 2025; and (4) contained complete bibliographic information eligible for subsequent analysis. Non-relevant publications and document types, including editorials, letters, conference abstracts, and book chapters, were excluded. All duplicate records were removed during database consolidation prior to eligibility screening.

Following database merging and deduplication, two reviewers independently screened the titles and abstracts of retrieved publications based on the predefined eligibility criteria. Any disagreements were resolved through consensus discussion. For all finally included publications, the following information was extracted: article title, authors, affiliations, country or region, keywords, source journal, publication year, citation metrics, and references. In this bibliometric study, the “population” was defined as published articles indexed in the selected databases, whereas the “outcomes” referred to a set of bibliometric indicators, including publication trends, collaborative patterns, and keyword evolution.

### 2.4 Database merging, deduplication, and data harmonization

To enhance the completeness and robustness of the dataset, this study integrated the search results from WoSCC and Scopus and used R to perform database merging, deduplication, and standardization. Scopus results were exported in .csv format, while WoSCC results were exported in.txt format. Given that the plain text format of WoSCC is more compatible with bibliometric analysis tools, this study adopted the WoSCC data structure as a reference to convert the format of Scopus records, ensuring consistency at the field level.

A multi-stage process was employed for duplicate identification. First, custom R scripts were utilized to conduct automatic matching and deduplication based on DOIs. For publications lacking DOIs, fuzzy matching was performed using title and author information to identify potential duplicate records. All candidate duplicates were subsequently reviewed manually to minimize the risk of erroneous deletion. Additionally, a random sample of the merged publications was audited to evaluate the accuracy of the deduplication process.

During the database integration process, data standardization was conducted to address inconsistencies in institutional information recording across the two databases. Since some institutional information in Scopus was presented as secondary affiliations, this study applied an Excel VBA program to map and unify such entries to the corresponding primary institutional names. Concurrently, the title field in Scopus was formatted to align with the record format of WoSCC. Finally, the researchers conducted a final consistency check and integration in Excel to ensure high uniformity across all records regarding authors, institutions, titles, and source information. After combining 1,217 records from WoSCC and 1,569 records from Scopus, an initial total of 2,786 merged records was obtained. A total of 1,164 duplicate records were identified and removed. Records without DOI information were not excluded solely due to missing DOI values; instead, potential duplicates were detected through title- and author-based fuzzy matching followed by manual verification. The remaining 1,622 unique publications were independently screened by two reviewers according to the predefined eligibility criteria. No additional publications were excluded during title and abstract screening; therefore, all 1,622 publications were included in the bibliometric analysis. Figure 1 presents the detailed literature identification, deduplication, screening, and inclusion process. The reporting of the study selection process followed the PRISMA 2020 guidelines, and the literature search strategy was reported in accordance with the PRISMA-S extension recommendations.

### 2.5 Bibliometric analysis and visualization

Microsoft Excel, VOSviewer, CiteSpace, and Bibliometrix were used to perform bibliometric analysis and visualization of the included literature. Excel was mainly applied for basic data sorting and statistical analysis, covering annual publication volume, cross-regional distribution, institutional and author distribution, and source journal distribution. VOSviewer was adopted to construct collaborative networks of countries/regions, institutions, and authors, as well as keyword co-occurrence networks. CiteSpace was utilized for keyword clustering, timeline evolution analysis, and emerging keyword detection. Bibliometrix was employed for thematic trend analysis, Bradford’s Law analysis, and supplementary bibliometric description (13–15). In all network visualizations, node size represents publication count or occurrence frequency, line thickness indicates connection strength, and different colors correspond to distinct clusters or temporal patterns (16). For CiteSpace analysis, node selection was performed using the g-index with a scaling factor of *k* = 20 to balance the inclusion of influential and newly emerging research items. Network construction adopted fractional counting, and association strength normalization was applied to standardize link weights across entities. The clustering resolution was set to 1.0. To enhance network interpretability, Pathfinder pruning and sliced network pruning were performed to eliminate redundant links while retaining the core network structure. For burst detection, the Kleinberg algorithm was applied with *α* = 2.0 and *γ* = 1.0 under two-state modeling, with a minimum burst duration of 2 years to identify emerging research trends.

## 3 Results

### 3.1 Publication output trend

**Figure 2:**
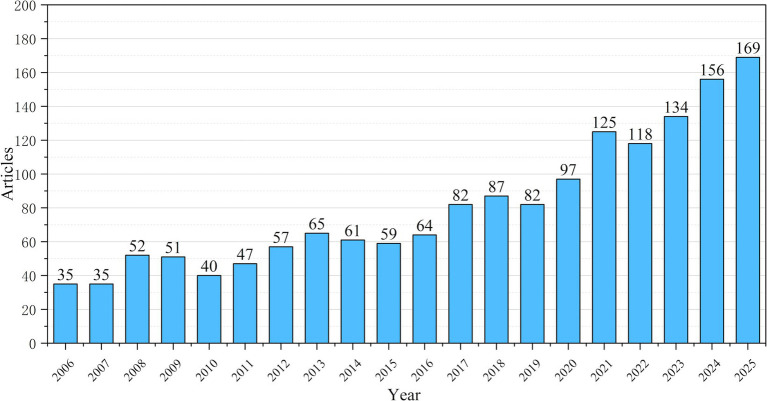
Annual scientific production from 2006 to 2025.

As shown in Figure 2, the annual number of publications in the field of diabetes and hearing loss shows an overall upward trend between 2006 and 2025, indicating that this topic has attracted increasing academic attention over the past 20 years. From 2006 to 2016, research in this area remained relatively stable, with annual publication volumes generally ranging between 35 and 65 articles. Although minor fluctuations occurred, an overall upward trend was already evident during this period. Since 2017, publication output in this field has entered a period of significant growth, reaching 82 articles in 2017 and 87 articles in 2018. Although the number of publications slightly declined to 82 in 2019, publication numbers continued to rise in the following years, increasing to 97 in 2020.

Notably, research activity in this field has intensified further since 2021, as annual publication volumes exceeded 100 for the first time and remained at a high level thereafter. The publication counts for 2021, 2022, and 2023 were 125, 118, and 134, respectively, further rising to 156 in 2024 and peaking at 169 in 2025, which represents the highest value across the entire study period. Overall, research on diabetes and hearing loss has exhibited an accelerating growth trend in recent years, reflecting that this topic has gradually become an important research direction within the interdisciplinary fields of otology, endocrinology, and geriatrics.

### 3.2 Country/region analysis

**Figure 3:**
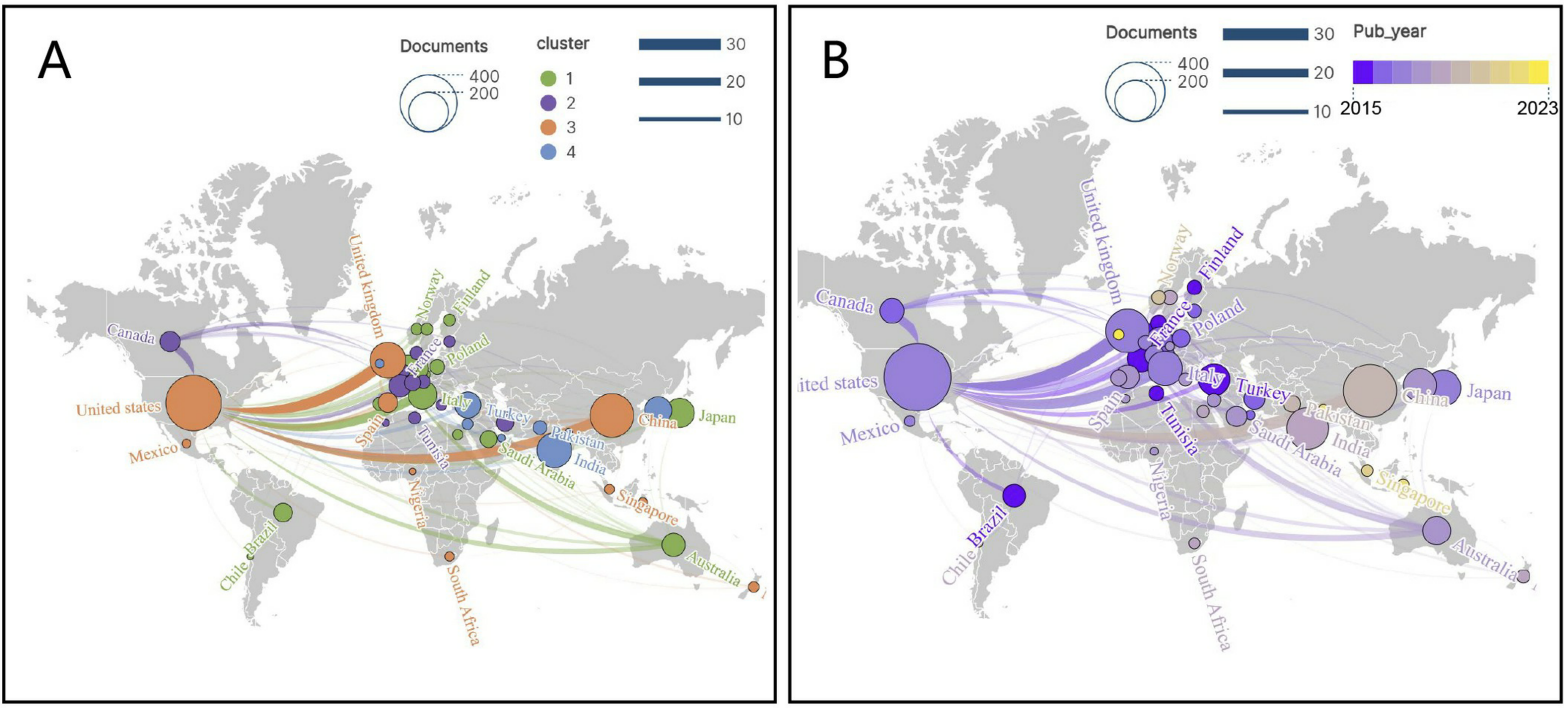
Collaboration network and overlay visualization of countries/regions in diabetes and hearing loss research. **(A)** Collaboration network of countries/regions. **(B)** Overlay visualization of countries/regions by publication year.

As shown in Figure 3A, research on diabetes and hearing loss has established a broad international collaboration network, with relevant studies covering numerous countries and regions across North America, Europe, Asia, Oceania, South America, and Africa. For network structure, countries and regions are closely connected, suggesting the cross-regional collaboration of this field. Overall, the national and regional collaboration network can be classified into four major clusters with abundant inter-cluster connections, which indicates that this research area has gradually formed a multi-centered international development pattern.

Among all participating countries and regions, the United States serves as the largest network node and sustains extensive, stable collaborative ties with many other countries, confirming its core position in this research field. Countries, including China, the United Kingdom, Japan, Italy, Canada, and Australia, present high publication activity and robust international collaboration capacity, acting as essential components of the global research network. In particular, the United States has built prominent collaborative partnerships with China, the United Kingdom, and various European and Asian countries, highlighting the vital contribution of intercontinental academic collaboration to research in this field.

Figure 3B presents the distribution of average publication dates across different countries and regions in this field. Overall, the United States, Canada, the United Kingdom, and several European countries are depicted in cooler colors. The colors indicate that these countries entered the research field early and served as key early contributors to studies on diabetes and hearing loss. In contrast, countries including China, Pakistan, Saudi Arabia, and Singapore are presented in relatively warmer colors, suggesting that these nations have experienced growing publication activity in recent years and are gradually emerging as new research forces in this field. These results indicate that research on diabetes and hearing loss has evolved from an early phase dominated by European and American countries into a global research network covering multiple regions, with Asian countries demonstrating a rising trend in academic contributions in recent years.

### 3.3 Analysis of institutions

**Figure 4:**
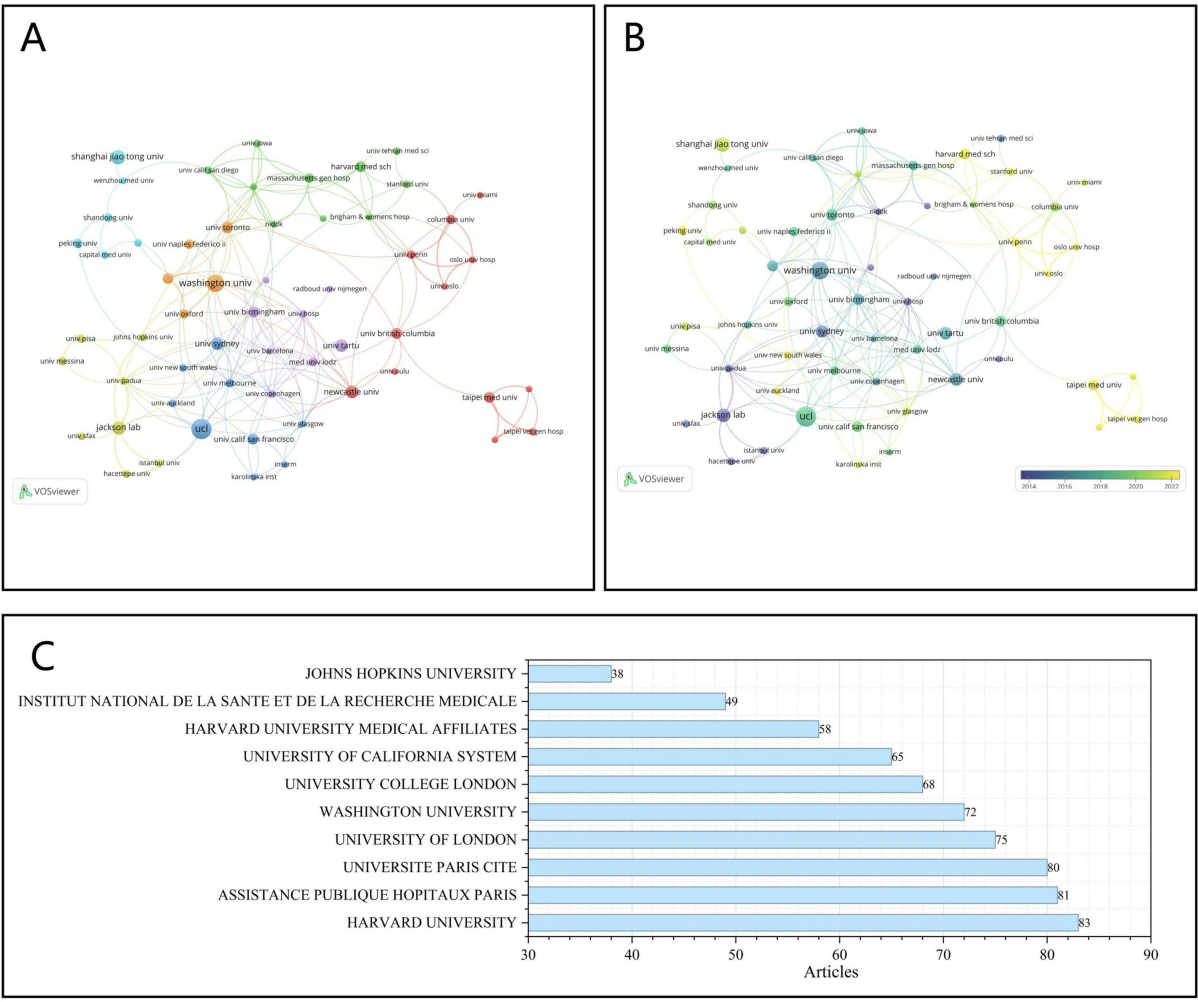
Analysis of institutions in diabetes and hearing loss research. **(A)** Institutional collaboration network. **(B)** Overlay visualization of institutions by publication year. **(C)** Top 10 most productive institutions.

Analysis of the institutional collaboration network reveals that research on diabetes and hearing loss has formed multiple interconnected clusters, yet it overall retains a distinct pattern of dominance by core institutions (Figure 4A). Among these institutions, Washington University, University College London (UCL), the University of Toronto, and Harvard Medical School are positioned at the center of the network and maintain connections with multiple nodes, suggesting that these institutions not only sustain high research activity but also play a vital role in connecting diverse collaborative clusters and promoting cross-institutional academic exchange. In contrast, several institutions are primarily distributed at the periphery of the network and only maintain local collaborative relationships, indicating that although a foundation for international collaboration has been established in this field, the collaborative landscape still presents a certain degree of centralization.

In terms of temporal evolution, notable differences exist in the active timeframes of different institutions in this field (Figure 4B). Overall, European and American institutions show earlier average publication dates, demonstrating that they initiated research on diabetes and hearing loss at an earlier stage and have sustained academic influence over a longer period. In comparison, several Asian institutions are marked by warmer color tones, reflecting later average publication dates and a more prominent growth in recent research activity. Among them, Taipei Medical University and Taipei Veterans General Hospital show relatively recent temporal characteristics, implying that they represent emerging research forces that have developed progressively in recent years. These temporal distribution differences indicate that the institutional landscape in this field is gradually expanding beyond the early dominance of Europe and the United States to incorporate more regions worldwide.

Further analysis of publication distribution indicates that the most productive institutions in this field are primarily concentrated in the United States, the United Kingdom, and France (Figure 4C). The top three institutions in terms of publication output are Harvard University (83 papers), Assistance Publique Hôpitaux de Paris (81 papers), and Université Paris Cité (80 papers), followed by the University of London, Washington University, and University College London. The gaps in publication output among these leading institutions are relatively small, suggesting that no single research center has established absolute dominance in this field; instead, the field’s development is collectively driven by multiple high-level research institutions. Notably, collaboration network analysis demonstrates that high publication output does not necessarily correspond to high collaborative centrality. While some institutions produce a large number of publications, their capacity to connect distinct collaborative groups remains relatively limited. Conversely, several network-core institutions exert a more pivotal influence on facilitating knowledge flow and cross-institutional collaboration. Overall, the institutional landscape of diabetes and hearing loss research is defined by dominant high-output institutions in Europe and the United States, core institutions that bridge collaborative networks, and the growing research activity of Asian institutions in recent years.

### 3.4 Analysis of authors

**Figure 5:**
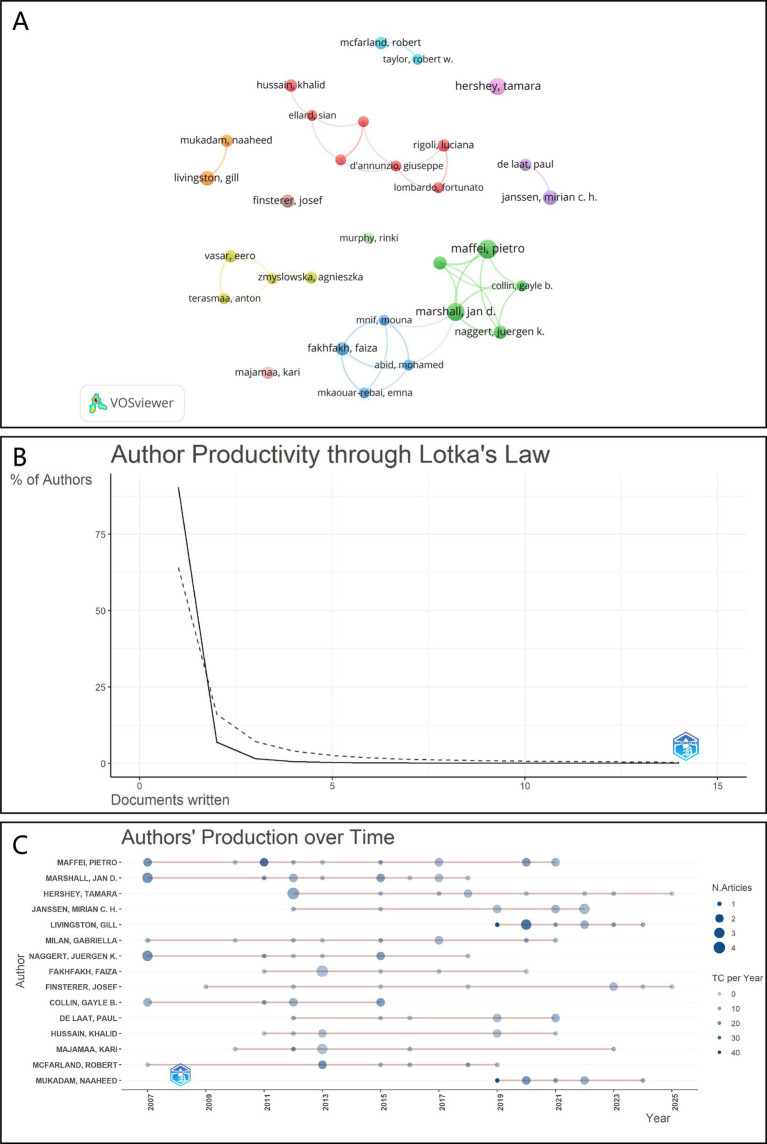
Analysis of authors. **(A)** Author collaboration network. **(B)** Author productivity through Lotka’s law. **(C)** Authors’ production over time.

Figure 5A illustrates the author collaboration network in this field, which presents distinct clustering alongside notable fragmentation. Although several small, clearly defined collaborative groups have formed in diabetes and hearing loss research, the overall network remains sparse, with limited inter-cluster connections and no highly integrated large-scale collaborative framework. The cluster centered on Pietro Maffei, Jan D. Marshall, Juergen K. Naggert, and Gayle B. Collin is the most prominent, with larger nodes and denser internal connections. It is therefore indicated that these authors are not only highly productive contributors but also play important organizational and bridging roles within the field. Similarly, the collaborative cluster consisting of Faiza Fakhfakh, Mohamed Abid, and Emma Mkaouar-Rebai shows evident internal collaboration features, while it has very few external ties with other research clusters in the network. Most remaining authors participate in paired or small-scale collaborative teams, while a number of authors occupy weakly connected or nearly isolated network positions. Collectively, the network reveals that author-level collaboration in this field is still predominantly restricted to stable small-team partnerships, with insufficient large-scale knowledge exchange and cross-group collaboration.

Figure 5B shows a typical Lotka-like distribution of author productivity, in which the proportion of authors declines sharply as publication output increases. The steep drop observed within the low-productivity range indicates that the vast majority of authors contributed only one or two publications, whereas only a small fraction maintained sustained multi-paper output. As indicated by this highly concentrated distribution, although the topic has attracted relatively broad academic participation, the pool of consistently productive core authors remains limited. More importantly, the predominance of low-output authors points to a structural pattern in which broad peripheral participation coexists with a relatively small core, indicating that the continuity and scholarly influence of the field still depend primarily on a small group of persistently active researchers.

Figure 5C further illustrates temporal disparities in author activity and the durability of scholarly contributions. Pietro Maffei and Jan D. Marshall demonstrate earlier research debuts, longer publication spans, and recurring output across multiple years, indicating that they rank among the most continuously active and influential authors in the field. By contrast, authors such as Tamara Hershey, Miriam C. H. Janssen, and Gill Livingston appear to have sustained relatively stable participation mainly over the past decade. Several other authors, however, feature publications confined to narrower time windows alongside relatively sparse bubble distributions, suggesting that their contributions were more closely tied to specific research stages, project cycles, or topic-centered collaborations. Taken together with annual publication counts and citation metrics, the timeline reveals that the field has not been steered by a single continuously dominant author; rather, it has evolved through a dynamic pattern in which a small group of long-term active scholars underpins research continuity while multiple intermittently active authors drive thematic expansion.

Figures 5A–C collectively indicate that the authorship landscape of diabetes and hearing loss research is defined by fragmented collaborative clusters, a restricted cohort of core authors, and a developmental trajectory primarily led by long-term active contributors. The fragmentation of the collaboration network and the concentration of productivity mutually reinforce one another, suggesting that, although several relatively stable research teams have taken shape, cross-team collaboration remains inadequate. Strengthening links between currently isolated author clusters, particularly through interdisciplinary cooperation spanning clinical medicine, genetics, neuroscience, and geriatric medicine, may advance knowledge integration, sustain research continuity, and boost the overall scholarly impact of this field.

### 3.5 Analysis of journals

**Figure 6:**
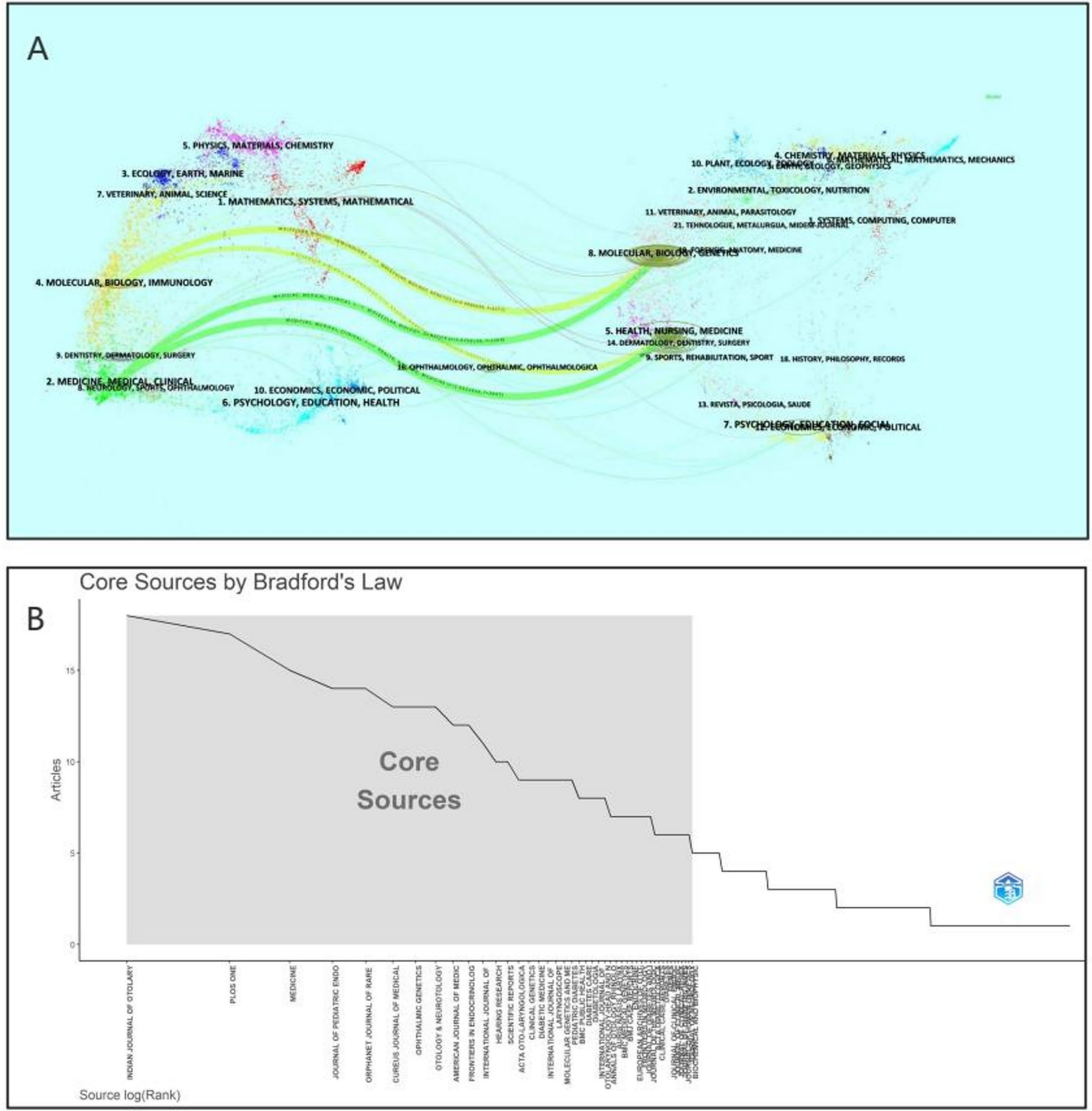
Analysis of journals. **(A)** Dual-map overlay of journals. **(B)** Core sources by Bradford’s law.

Figure 6A delineates the disciplinary structure and knowledge-flow pattern of research on diabetes and hearing loss. The citing journals on the left are concentrated primarily in Medicine, Clinical Medicine, Molecular Biology, and Immunology domains, whereas the cited journals on the right are distributed mainly across Molecular Biology, Genetics, Health Nursing, and Medicine. This distribution indicates that the field exhibits a distinctly interdisciplinary character at both the knowledge production and knowledge absorption levels. More importantly, the most prominent citation trajectories extend from clinically oriented journals to journals covering molecular biology, genetics, and broader health sciences, suggesting that this research area originates largely from clinical questions while remaining strongly grounded in mechanistic interpretations, genetic evidence, and integrated medical knowledge. In this sense, the map reveals a relatively clear knowledge flow pattern moving from clinical observation to mechanistic explanation and, ultimately, comprehensive medical integration, underscoring the translational nature of the field.

Figure 6A also suggests that the intellectual input of this research area is not confined to a single medical specialty. Instead, it presents a convergent structure where clinical disciplines, basic biosciences, and general health sciences interact closely. These cross-domain citation links imply that the field has moved beyond descriptive evaluations of auditory impairment in diabetic populations and has increasingly incorporated genetic backgrounds, molecular pathways, and systemic pathological mechanisms into its analytical framework. Accordingly, the map captures not only disciplinary distribution but also an evolving research logic defined by clinical question-driven inquiry, mechanism-based interpretation, and multidisciplinary evidence integration.

Bradford’s law analysis was conducted via the Bibliometrix R package, with journals automatically categorized into three zones (core, zone 1, and zone 2) according to their ranked productivity distribution. Figure 6B further illustrates journal concentration patterns based on Bradford’s law. The shaded core zone contains the primary publication outlets for this field, indicating that a large proportion of relevant literature accumulates within a small set of highly specialized journals. Top-ranked journals published approximately 18 articles, after which publication volumes declined progressively alongside journal rank; outside the core zone, per-journal article counts fell rapidly to fewer than 5, forming a distinct long-tail distribution. This result suggests that, while research on diabetes and hearing loss spans otology, endocrinology, genetics, and general medicine, scholarly outputs are not evenly dispersed across journals but instead rely heavily on a relatively small collection of core outlets.

The composition of the core-source set, including Indian Journal of Otolaryngology, PLOS ONE, Medicine, Journal of Pediatric Endocrinology, Orphanet Journal of Rare Diseases, Cureus, Ophthalmic Genetics, Otology and Neurotology, American Journal of Medical Genetics, and Frontiers in Endocrinology, further corroborates this interpretation. These journals cover otolaryngology, endocrinology, genetics, and general medicine, which aligns closely with the interdisciplinary knowledge base identified via the dual-map overlay. Thus, while this field draws upon diverse disciplinary traditions, its scholarly communication has gradually converged toward a stable set of specialist and multidisciplinary journals, reflecting the progressive maturation of its research community and publication channels.

Figures 6A,B collectively demonstrate that research on diabetes and hearing loss is defined by prominent interdisciplinarity and an evident concentration of core publication outlets. On the one hand, citation trajectories reveal that the field is rooted in clinical medicine and continuously absorbs theoretical and empirical evidence from molecular biology, genetics, and health sciences. On the other hand, the formation of a core journal set indicates that this research topic is transitioning from an early phase of scattered exploration to a more consolidated and standardized stage of knowledge production and dissemination. Overall, these findings confirm that the field has established a preliminary academic framework centered on clinically oriented research questions, supported by mechanistic investigations, and disseminated through a stable core of academic journals.

### 3.6 Analysis of the references

**Figure 7:**
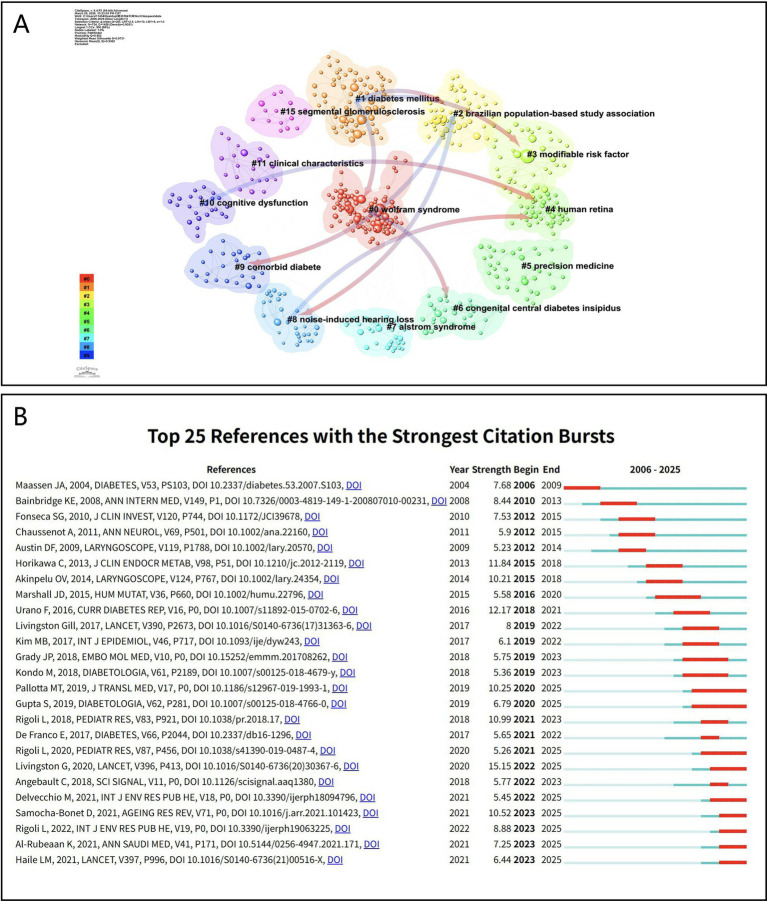
Analysis of references. **(A)** Co-citation clustering network of references. **(B)** Top 25 references with the strongest citation bursts.

The analysis of references further unpacks the foundational knowledge and evolving research frontiers of studies concerning diabetes and hearing loss. Figure 7A presents the co-citation clustering structure of cited references; the whole network forms several knowledge clusters with relatively distinct boundaries, implying that a relatively stable research foundation has been constructed within this field. The primary clusters include #0 Wolfram syndrome, #1 diabetes mellitus, #3 modifiable risk factors, #5 precision medicine, #7 Alström syndrome, #8 noise-induced hearing loss, #9 comorbid diabetes, and #10 cognitive dysfunction. As indicated by the above-mentioned clusters, the knowledge base for diabetes and hearing loss research extends beyond discussions of disease correlation and simultaneously covers multiple dimensions, such as genetic syndromes, risk factor identification, comorbidity burden, cognitive function, and precision medicine. Among these, Wolfram syndrome and diabetes mellitus take up central positions and exhibit evident connections with multiple other clusters. It is therefore revealed that investigations into genetic syndromes and the clinical phenotyping of diabetes together form the core knowledge pillars in this field.

In terms of cluster content, the early knowledge base primarily focused on Wolfram syndrome, Alström syndrome, congenital or monogenic diabetes, and mitochondrial-related diseases, which aligns with the sustained activity of keywords such as “monogenic diabetes,” “mitochondrial disease,” and “WFS1 gene” in the aforementioned keyword analysis. Meanwhile, the emergence of clusters including “modifiable risk factors,” “cognitive dysfunction,” and “precision medicine” indicates that the research focus in this field has gradually shifted in recent years from rare genetic disease models to broader clinical risk assessment, population management, and personalized intervention strategies. In other words, the co-citation structure of references reveals that the knowledge framework of this field has gradually transitioned from the early syndrome-focused and mechanistic research to a comprehensive research model integrating epidemiology, clinical phenotypes, and precision medicine.

Changes in research frontiers are more clearly reflected in the emergence analysis of references. Figure 7B lists the top 25 references with the highest emergence intensity. Although their emergence spans the entire research period, the core focused literature varies distinctly across different stages. The early emerging literature mainly covered diabetes-related cognitive and metabolic abnormalities, syndrome-based diseases, and clinical observations in audiology, including the studies conducted by Maassen et al., Bainbridge et al., Fonseca et al., and Austin et al. This finding suggests that early research paid more attention to diabetes-associated neurological changes, metabolic disorders, and corresponding auditory manifestations. In the middle research stage, the emerging studies from Horikawa, Akinpelu, Urano and other scholars demonstrate that the research focus has gradually expanded to the epidemiology of hearing loss, clinical risk factors, and the underlying mechanisms of diabetes-related complications.

More notably, the number of emerging references has increased significantly in recent years up to 2025, with research topics increasingly concentrated on aging, monogenic diabetes, mitochondrial diseases, epidemiological associations, and environmental exposure factors. For instance, studies by Pallotta (2019), Gupta (2019), Rigoli (2020, 2022), Livingston (2020), Delvecchio (2021), Samocha-Bonet (2021), and Haile (2021) have maintained strong emergence intensity in recent years, indicating that the research frontiers in this field are further expanding toward genetic profiling, aging-related problems, and public health-level risk identification. Among these studies, Livingston (2020) presented the highest emergence intensity of 15.15, which indicates that high-impact research on cognitive decline and aging has begun to profoundly shape the development direction of research on diabetes and hearing loss.

Combining the results of co-citation clustering and emerging reference analysis reveals that the knowledge base for research on diabetes and hearing loss is initially established primarily on genetic syndromes, mitochondrial abnormalities, and observational studies, whereas the current research frontier is gradually shifting toward research frameworks focused on modifiable risk factors, cognitive impairment, aging populations, and precision medicine. This evolutionary trend not only indicates that the research focus in this field is shifting from relatively specific disease models to broader clinical and public health issues, but also suggests that future research should further strengthen mechanistic research, risk stratification, and interdisciplinary integration.

### 3.7 Keywords analysis

**Figure 8:**
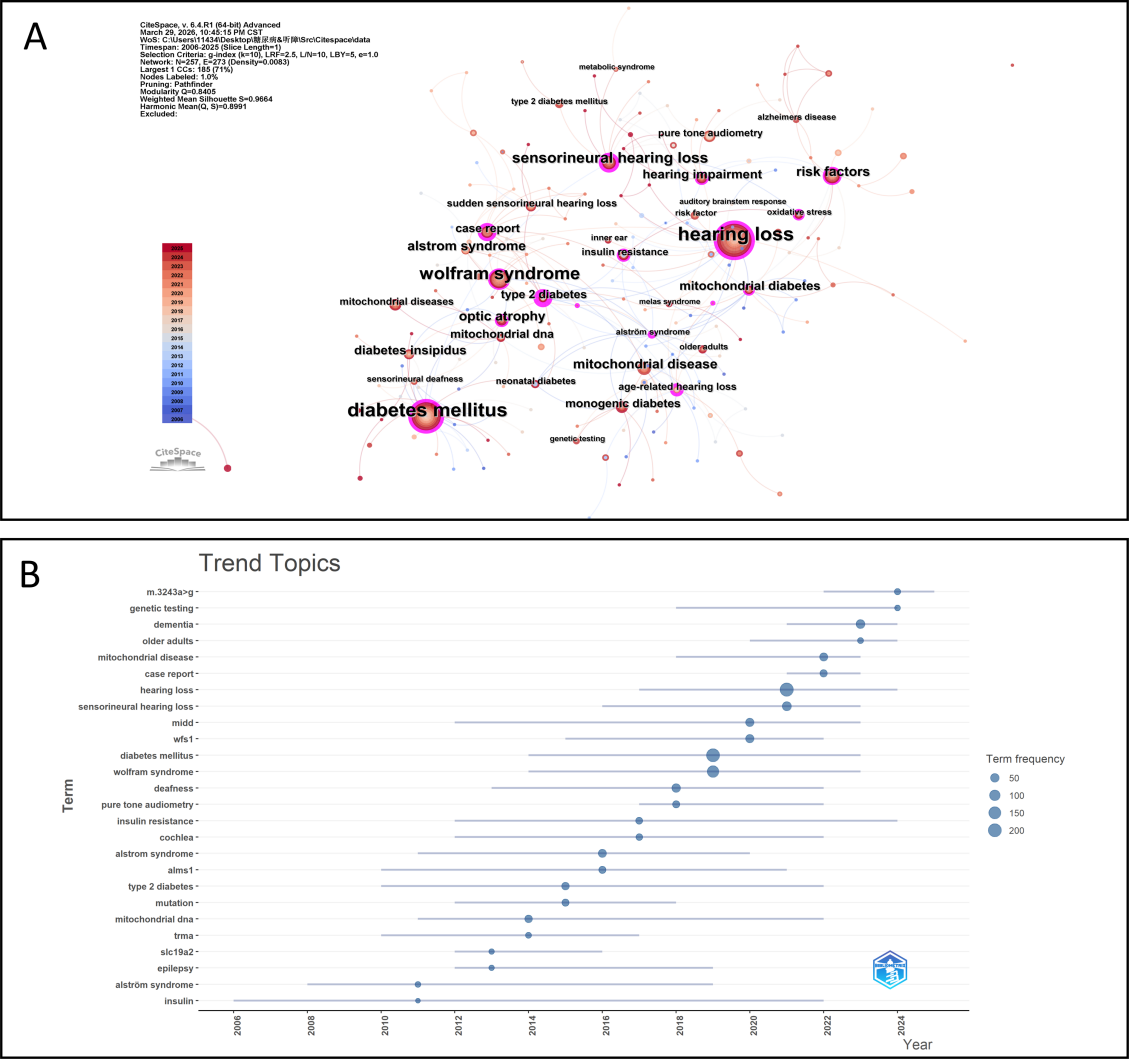
Analysis of keywords. **(A)** Keyword co-occurrence network. **(B)** Trend topics.

Integrated keyword analysis reveals a relatively coherent knowledge structure in the research field of diabetes and hearing loss. The co-occurrence network identifies hearing loss, diabetes mellitus, Wolfram syndrome, sensorineural hearing loss, and mitochondrial disease as central nodes with extensive connections to surrounding terms, indicating that these topics constitute the core conceptual basis of the field (Figure 8A). Among these core nodes, hearing loss occupies the most central position and is closely associated with risk factors, hearing impairment, pure tone audiometry, type 2 diabetes, and insulin resistance, suggesting that the research field has evolved from descriptive association studies to research focusing on risk evaluation, audiological assessment, and metabolic mechanisms.

Trend-topic analysis demonstrates a clear temporal shift in research focus (Figure 8B). Early studies focused on genetically and metabolically relevant topics, including insulin, Alström syndrome, SLC19A2, TRMA, and mitochondrial DNA. This research focus later expanded to more clinically oriented topics, such as type 2 diabetes, cochlea, insulin resistance, pure tone audiometry, and deafness. In recent years, the rising prominence of research on older adults, dementia, genetic testing, and the m.3243A > G variant indicates growing academic attention to aging, cognitive impairment, and precision diagnostic approaches.

**Figure 9:**
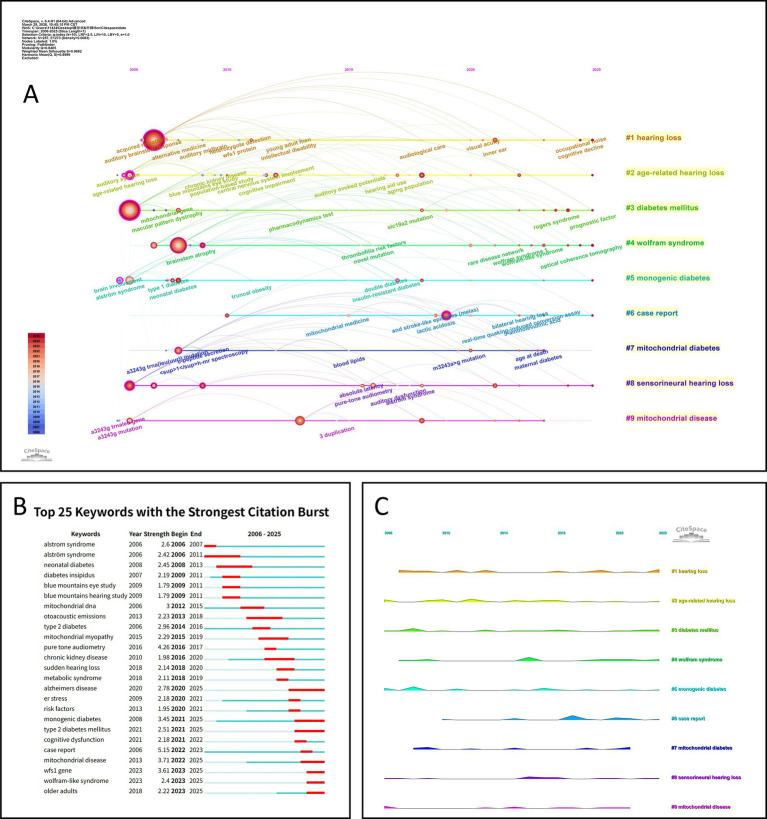
**(A)** Timeline view of keyword clusters. **(B)** Temporal distribution of major keyword clusters. **(C)** Top 25 keywords with the strongest bursts.

The timeline clustering map further validates this thematic evolution pattern (Figure 9A). The major research clusters include hearing loss, age-related hearing loss, diabetes mellitus, Wolfram syndrome, monogenic diabetes, mitochondrial diabetes, sensorineural hearing loss, and mitochondrial disease. Among these clusters, hearing loss and diabetes mellitus have persisted throughout the entire research period, demonstrating their role as the long-term thematic backbone of this research field. In contrast, clusters related to Wolfram syndrome, monogenic diabetes, and mitochondrial diabetes reflect the sustained importance of hereditary syndromes and mitochondrial disorders in mechanistic research.

Thematic intensity analysis indicates that stable core research topics coexist with episodic thematic research directions (Figure 9B). Hearing loss, age-related hearing loss, and diabetes mellitus remain consistently active research hotspots over time, whereas case reports and mitochondrial diabetes research exhibit more stage-specific fluctuating trends. This evolutionary pattern suggests that the development of this research field is driven by the dual effects of persistent core research topics and intermittent breakthroughs in clinical and mechanistic research areas.

Burst-keyword detection further identified the frontier directions of the field (Figure 9C). Early burst terms, including Alström syndrome, neonatal diabetes, diabetes insipidus, the Blue Mountains Eye Study, and the Blue Mountains Hearing Study, reflect an initial research emphasis on syndromic disorders and epidemiological observation. Later burst terms shifted toward mitochondrial DNA, otoacoustic emissions, type 2 diabetes, pure tone audiometry, chronic kidney disease, and sudden hearing loss, indicating a research expansion toward hearing assessment, metabolic comorbidity, and clinical phenotype characterization. Recent burst terms that persist through 2025, including Alzheimer’s disease, monogenic diabetes, type 2 diabetes mellitus, mitochondrial disease, the WFS1 gene, wolfram-like syndrome, and older adult populations, suggest that current research increasingly focuses on genetic mechanisms, mitochondrial dysfunction, aging processes, and cognitive decline.

Overall, keyword-based analyses indicate that research on diabetes and hearing loss has evolved from early syndrome-focused and association-oriented exploration into an integrated research framework. This framework is characterized by sustained attention to hearing loss and diabetes mellitus, continuous research interest in hereditary and mitochondrial mechanisms, and a growing focus on aging, cognition, and precision medicine.

## 4 Discussion

### 4.1 General characteristics and global research structure

This systematic bibliometric review demonstrates that research on diabetes and hearing loss has expanded steadily from 2006 to 2025, with a notably accelerated growth after 2017 and continuous development after 2021. This temporal trend likely reflects several converging developments. First, the global prevalence of diabetes has been continuously rising. Thus, the clinical and public health significance of diabetes-associated complications has been enhanced. Second, population aging has increased the number of individuals concurrently affected by metabolic disorders, auditory deterioration, and cognitive vulnerability. Third, advances in audiological assessment, genetic testing, mitochondrial research, and large-scale health databases have enabled investigations into diabetes-related auditory dysfunction from epidemiological, clinical, and mechanistic perspectives (8, 17). Accordingly, the growing publication output reflects not only rising academic interest but also the gradual expansion of this research field from rare syndromic disorders to prevalent metabolic and age-related hearing impairment. Nevertheless, growth in publication volume does not necessarily correspond to improved evidence quality, as most existing studies remain observational and exhibit heterogeneity in population selection, hearing assessment, diabetes definition, and confounding factor control.

The country and regional analyses show that the United States occupies the most central position in the international collaboration network, with the United Kingdom, Canada, Italy, Japan, China, and Australia serving as important components of the global research structure. The leading position of the United States may be partly explained by its long-established epidemiological cohorts, robust research infrastructure in endocrinology, audiology, genetics, and aging, as well as extensive cross-institutional collaboration. Its central network position suggests that its influence is not merely limited to publication output, but also includes a bridging role in connecting national research groups that would otherwise remain separated. By contrast, according to the recent average publication years observed for China, Pakistan, Saudi Arabia, and several other Asian countries, these regions have entered a phase of accelerated growth. This trend may be associated with the rising diabetes burden, rapid demographic aging, improved access to audiological services, and the expansion of population-based and hospital-based research databases. However, the network still presents a marked imbalance in global participation. Countries from sub-Saharan Africa, parts of Southeast Asia, and other low- and middle-income regions are relatively weakly represented, despite facing substantial diabetes burdens and limited access to hearing care. Such geographic imbalance may reduce the external validity of current findings, because the interaction between diabetes and hearing loss may vary according to ethnicity, environmental exposure, healthcare accessibility, disease management, and socioeconomic conditions. The institutional findings further demonstrate that scientific productivity and collaboration centrality are not equivalent indicators. Harvard University, Assistance Publique-Hôpitaux de Paris, and Université Paris Cité are among the most productive institutions, whereas Washington University, University College London, and the University of Toronto appear to occupy more central positions in the collaboration network. This distinction is important because publication volume reflects tangible research output, whereas network centrality reflects the ability to connect institutions, facilitate knowledge exchange, and support cross-regional collaboration. Institutions with high output but relatively limited network connectivity may contribute substantially within a specific research tradition, while institutions with stronger bridging roles may exert greater influence on the diffusion of methods, concepts, and collaborative practices. The growing activity of Taipei Medical University and Taipei Veterans General Hospital also suggests that the institutional center of the field is gradually expanding beyond its traditional concentration in Europe and North America. Future multicenter studies should build on this trend by linking highly productive institutions with emerging research centers, thereby improving population diversity, methodological comparability, and international knowledge transfer within this research field. At the author level, the collaboration network remains fragmented despite the presence of several stable research groups. The clusters centered on Pietro Maffei, Jan D. Marshall, Juergen K. Naggert, Gayle B. Collin, Faiza Fakhfakh, Mohamed Abid, and Emma Mkaouar-Rebai illustrate how much of the field has developed through relatively small, topic-specific teams. These groups have made sustained contributions, particularly regarding genetic syndromes, mitochondrial disorders, and rare forms of diabetes associated with hearing loss (18, 19). However, the limited connections between clusters suggest that knowledge production remains compartmentalized across genetics, endocrinology, audiology, neurology, and geriatric medicine. The Lotka-like distribution of author productivity further indicates that most researchers have contributed only one or two publications, while a small number of authors have maintained long-term research activity. This pattern is common in emerging interdisciplinary fields but may also limit research continuity, replicability, and methodological standardization. Strengthened cross-team collaboration could facilitate the integration of mechanistic findings on rare diseases with large-scale epidemiological evidence and clinically oriented screening research. The journal analysis confirms that diabetes-related hearing loss has developed as a highly interdisciplinary research area. The dual-map overlay demonstrates that clinically oriented research draws heavily on molecular biology, genetics, immunology, and broader health sciences, indicating that the field is driven by clinical questions while being increasingly elucidated through (20, 21). Bradford’s law analysis further shows that a substantial proportion of publications are concentrated in a relatively small set of core journals covering otolaryngology, endocrinology, genetics, and general medicine. This publication concentration suggests that the field has begun to establish relatively stable academic communication channels. Meanwhile, the disciplinary diversity of these core journals reflects the absence of a single dominant disciplinary domain for diabetes-related hearing loss research. Future progress will therefore rely on closer integration between clinical audiology, metabolic medicine, genetics, epidemiology, and aging research, as well as the development of more standardized definitions, outcome measures, and reporting protocols.

### 4.2 Research hotspots and trends

Keyword analysis and burst detection reveal that research on diabetes and hearing loss has undergone a clear thematic evolution over the past two decades. Rather than remaining static the field has progressively shifted from an initial focus on rare syndromic and mitochondrial disorders toward broader (4, 22) and more recently toward aging-related mechanisms cognitive outcomes and precision medicine-oriented research. This transition indicates that the field is moving beyond disease-specific characterization toward clinically oriented risk stratification and translational applications.

In the early stage of development, research attention was largely concentrated on rare genetic syndromes and mitochondrial dysfunction. These topics, represented by Wolfram syndrome, mitochondrial disease, and related monogenic diabetes phenotypes, reflect initial efforts to understand the mechanistic basis of diabetes-associated auditory impairment (23). Such disorders serve as important biological models because they simultaneously involve metabolic dysfunction, neurodegeneration, and sensorineural hearing loss. The sustained emergence of genes such as WFS1 and mitochondrial variants such as m.3243A > G further indicates that mitochondrial energy failure and genetic susceptibility remain central mechanistic foundations of this field. This stage primarily contributed to the establishment of biological plausibility linking metabolic dysfunction and cochlear injury (24, 25).

With the expansion of epidemiological datasets and clinical cohorts, research focus has gradually shifted toward population-based risk assessment and metabolic associations. Keywords such as type 2 diabetes, insulin resistance, risk factors, and hearing impairment reflect growing scholarly interest in determining whether dysglycemia and insulin resistance increase susceptibility to auditory decline (26). This shift indicates that the field has moved beyond establishing basic associations toward quantifying relevant risks and identifying susceptible populations. Mechanistically, this transition is supported by accumulating evidence implicating microvascular damage, oxidative stress, chronic inflammation, and neuronal injury induced by long-term metabolic dysregulation (27, 28). These pathways collectively provide a biological explanation for the observed epidemiological associations.

In parallel, audiological assessment and early detection strategies have become increasingly prominent research directions. The emergence of terms including pure tone audiometry, otoacoustic emissions, cochlea, and subclinical hearing loss indicates a research shift from clinically evident hearing impairment toward the early stages of auditory dysfunction (26, 29). This development reflects the recognition that cochlear damage induced by diabetes may occur prior to the appearance of overt hearing symptoms. Among existing diagnostic tools, otoacoustic emissions offer higher sensitivity for detecting early outer hair cell dysfunction, while pure tone audiometry remains the standard clinical assessment method. This methodological evolution highlights the growing emphasis on early screening and preventive intervention strategies within the field.

More recently, keyword evolution demonstrates that research on diabetes-related hearing loss has increasingly intersected with aging, cognitive decline, and neurodegenerative disease. The emergence and sustained prevalence of keywords such as Alzheimer’s disease, dementia, and older adults suggest that researchers are exploring the shared pathological mechanisms underlying metabolic dysfunction, auditory decline, and cognitive impairment (30, 31). These associations are likely mediated by overlapping biological pathways, including microvascular injury, mitochondrial dysfunction, neuroinflammation, and systemic metabolic dysregulation (31, 32). This expanding research direction reflects a broader conceptual shift, in which hearing loss is no longer regarded as an isolated sensory disorder but as a component of systemic aging-related decline, with potential implications for dementia risk stratification (33).

Taken together, the evolution of research hotspots demonstrates a progressive transition from rare disease-oriented mechanistic studies to epidemiological risk assessment, followed by growing attention to early diagnosis and ultimately to aging-related cognitive outcomes. This research trajectory reflects the maturation of this field from disease-specific investigations to an integrated research framework that combines metabolic, auditory, and neurocognitive perspectives, with increasing emphasis on translational and precision medicine applications (4, 34, 35).

### 4.3 Strengths and limitations

This study possesses several methodological strengths. First, it integrates two major citation databases, the WoSCC, which jointly deliver broad multidisciplinary coverage and boost the comprehensiveness of bibliometric retrieval. Compared with single-database analyses, this dual-database strategy lowers selection bias and strengthens the robustness of the constructed collaboration networks and keyword co-occurrence structures. Second, this study adheres to the PRISMA 2020 reporting framework and further complies with PRISMA-S guidelines for search strategy design, thus raising the transparency and reproducibility of the literature screening process. Third, multiple complementary bibliometric tools, including VOSviewer, CiteSpace, and Bibliometrix, were utilized to capture distinct dimensions of the research structure, such as collaboration networks, thematic clustering, and temporal evolution patterns. Joint application of these tools offers a more comprehensive and triangulated depiction of the research landscape.

Despite the above strengths, several limitations of this work require acknowledgment. First, all analyses were confined to the WoSCC databases; accordingly, papers indexed solely in PubMed, Embase, or regional databases were excluded. This setup may result in partial omission of relevant literature, especially research published in non-indexed or region-focused journals. Second, only English-language publications were retained to guarantee consistent bibliographic field extraction and unified keyword standardization across databases. Nevertheless, this practice creates language bias, which may undercount research outputs from non-English-speaking regions and potentially alter the observed geographic distribution of academic publications.

Third, bibliometric analysis inherently relies on the completeness and structure of bibliographic metadata, alongside algorithm parameter configurations embedded in different visualization tools. Discrepancies in author name disambiguation, institutional normalization, and citation indexing may generate minor measurement bias, even after manual data cleaning and harmonization steps. Furthermore, though DOI matching and manual validation were implemented for deduplication, residual duplicate or misclassified records cannot be fully eliminated. Lastly, bibliometric approaches excel at identifying structural patterns, collaboration frameworks and research trends, yet they cannot directly evaluate the methodological quality or clinical validity of individual studies. For this reason, the results of this study should be regarded as a macroscopic mapping of existing literature rather than a synthesis of evidence strength.

Taken together, these limitations do not undermine the overall validity of the detected trends, yet they must be taken into account when interpreting the geographic distribution, thematic evolution, and collaboration structures presented in this study.

## 5 Conclusion

This study performs a systematic bibliometric analysis of research on diabetes and hearing loss covering the period from 2006 to 2025. The findings indicate that the publication output of this field has maintained a sustained upward trend over the past two decades, with research activity further intensifying in recent years, which suggests growing academic and clinical interest in this interdisciplinary research topic.

The United States occupies a core position in the global research collaboration network, while major universities and medical research institutions across Europe and North America serve as key hubs for knowledge production. Meanwhile, the rising academic activity of Asian countries and relevant institutions highlights their gradually expanding contributions and influential roles in this research field.

Keyword analysis reveals that research hotspots have evolved from early-stage focuses on syndromic disorders and mitochondrial diseases to a broader research scope covering epidemiological associations risk factor identification aging populations cognitive impairment and genetics- and precision medicine-based research. Overall research on diabetes and hearing loss is shifting from relatively narrow disease-specific exploration toward a more integrated clinically oriented and translational research paradigm.

Future studies should further strengthen cross-national, cross-institutional and interdisciplinary collaboration, while placing greater emphasis on high-risk population identification, mechanistic elucidation, early screening, and the development of precision intervention strategies.

## Data Availability

The raw data supporting the conclusions of this article will be made available by the authors, without undue reservation.
